# *4th International Symposium on Sensor Science* (I3S2015): Conference Report

**DOI:** 10.3390/s150924458

**Published:** 2015-09-23

**Authors:** Peter Seitz, Debbie G. Senesky, Michael J. Schöning, Peter C. Hauser, Roland Moser, Hans Peter Herzig, Assefa M. Melesse, Patricia A. Broderick, Patrick Thomas Eugster

**Affiliations:** 1 Hamamatsu Photonics Innovation Center Europe, Solothurn 4500, Switzerland; 2 Innovation and Entrepreneurship Lab, ETH Zürich, Zürich 8092, Switzerland; 3 Institute of Microengineering, EPFL Neuchâtel, Neuchâtel 2002, Switzerland; 4 Stanford University, Stanford, CA 94305, USA; E-Mail: dsenesky@stanford.edu; 5 Aachen University of Applied Sciences, 52066 Aachen, Germany; E-Mail: schoening@fh-aachen.de; 6 University of Basel, Basel 4056, Switzerland; E-Mail: Peter.Hauser@unibas.ch; 7 Alstom, Baden 5400, Switzerland; E-Mail: roland.moser@power.alstom.com; 8 EPFL Lausanne, Lausanne 1015, Switzerland; E-Mail: hanspeter.herzig@epfl.ch; 9 Florida International University, Miami, FL 33199, USA; E-Mail: melessea@fiu.edu; 10 City College of New York, New York, NY 10031, USA; E-Mail: broderick@med.cuny.edu; 11 Purdue University, West Lafayette, IN 47907, USA; E-Mail: peugster@cs.purdue.edu

## 1. Introduction

An international scientific conference was sponsored by the journal *Sensors* under the patronage of the University of Basel. The 4th edition of the *International Symposium on Sensor Science* (I3S2015) ran from 13 to 15 July 2015 in Basel, Switzerland. It comprised five plenary sessions and one morning with three parallel sessions. The conference covered the most exciting aspects and the latest developments in sensor science. The conference dinner took place on the second evening of the conference. The I3S2015 brought together 170 participants from 40 different countries.

The next edition of the I3S2016 conference will be held in Durham, NH, USA, 17–22 July 2016.

## 2. Session 1: Sensor Breakthroughs

Session Chair: **Debbie G. Senesky**

The first session of the symposium provided an update on recent sensor breakthroughs. The keynote speaker, **R. Horisberger**, impressed the attendees with a detailed description of the use of supreme sensing to answer the ultimate questions of physics. **C. Fattinger** presented the use of focal molography to realize coherent detection of biomolecular interactions for a variety of biological applications. **V.M. Mirsky** presented the creation of ultrasensitive sensors by leveraging surface plasmon resonance (SPR). **L.S. Marcus** discussed trace gas sensing and standoff detection of solid materials using photoacoustic spectroscopy-based sensor platforms. **T. Wagner** reviewed the progress in creating a fully-integrated (bio-)chemical sensor platform using light-addressable potentiometric sensors. The session closed with a talk by **E. Katz** on binary operating biosensors based on biocomputing systems. It was a lively session with active discussions that provided new insights for the sensor science community.

## 3. Session 2: Biosensors

Session Chair: **Michael J. Schöning**

This session has been focussing on recent developments in the field of different types of biosensors as well as some nanosensor-based applications. Besides physical sensors and chemical sensors, biosensors belong to the third class of sensing devices with strongly increasing interest within the last 20 years. There are even several (worldwide) conferences purely addressing this topic year by year. The biosensors session offered a keynote lecture, given by **H. Ju** (China), two invited lectures by **A. Star** (USA) and **S. Kolev** (Australia), and five selected presentations by **T. Yoetz** (Isreal), **L. Francis** (Belgium), **G. Zabow** (USA), **A. Poghossian** (Germany) and **M. Packirisamy** (Canada).

All presentations have been driven by application-oriented research activities―Coming from pure “academic” research into the “real” market: **H. Ju**, for instance, gave an overall survey on a multitude of possible signal amplification strategies for biosensing, also covering important nano-aspects in optimizing intrinsic biosensor features. **A. Star** and **S. Kolev** discussed on the one hand different chemical and biological sensor approaches with carbon nanostructures, and on the other hand, easy to fabricate and cheap, disposable paper-based microfluidic sensors for environmental monitoring and analysis. Further aspects in this session have been covering biosensors for the detection of proteins by means of nanostructured integrated optofluidic chips (**M. Packirisamy**), micro- to nanoscale magnetic-type (bio)sensors offering opportunities similar to those of fluorescence-type optical biosensors but operating at radio-frequency wavelengths (**G. Zabow**), semiconductor-based field-effect biosensors for the label-free detection of single- and double-stranded DNA molecules (**A. Poghossian**), amperometric biosensors utilizing bacteria for the screening of cytochrome P450 inhibitors (**T. Yoetz**) and an integrated capacitive biosensor array for the selective and real-time detection of whole bacterial cells with a high sensitivity.

In conclusion, it was a very active session including very fruitful discussions. All main transducer principles of biosensors were presented, *i.e.*, there was a representative cross section of running biosensor activities from all over the world.

## 4. Session 3: Chemical Sensors

Session Chair: **Peter C. Hauser**

The session on chemical sensors was highly diverse in terms of sensing principles, as well as applications. **T. Yoshinobu**, the keynote lecturer, showed that it is possible to obtain spatially-resolved measurements with an array of potentiometric sensors, which can be individually addressed with a light beam. **D. Diamond** stressed that chemical sensors often are best employed as part of an analytical system based on microfluidics. **R. Seitz** discussed optical sensing of metals with membranes containing fluorophores. **K. Bonnot** reported on the calorimetric determination of explosives, *i.e.*, methods based on their combustion in porous materials. The second speaker from Japan, **K. Mitsubayashi**, brought us back to spatially-resolved measurements, this time on the determination of ethanol vapors (e.g., in exhaled breath) by picturing the chemiluminescence obtained on a mesh impregnated with the reaction mixture. **J. Stiens** discussed the use of THz waves in sensing, while **E. Gallegos-Arellano** employed the more conventional IR range for the determination of CO and CO_2_. The last speaker, **F. Josse**, described the employment of surface acoustic waves for the determination of aromatic compunds such as benzene using appropriate membranes with affinity to the analysts. All in all, it was a very satisfying session with something to hold everybody’s interest.

## 5. Session 4: Extreme Sensing

Session Chair: **Roland Moser**

This session offered a series of amazing accomplishments of sensing in extreme environments or sensing with extreme performance. The first talk given by **D.G. Senesky** (Stanford) covered micro- and nano-scale sensors for extreme harsh environments: By using wide-bandgap semiconductors, such as silicon carbide or gallium nitride, complete sensor systems can be built that can reliably acquire and transmit data at ambient temperatures of a scorching 600 °C or even more. This exciting opening speech of the session was followed by **M.J. Schöning**’s (FH Aachen) description of thin-film sensors for monitoring aseptic food processes. This requires extremely robust sensor types withstanding the highly-corrosive effects of the employed potent sterilization gas hydrogen peroxide (H_2_O_2_). The next presentation on a high-sensitivity NO, NO_2_ and NH_3_ high electron mobility transistor (HEMT)-based sensor for diesel exhaust systems was contributed by **Y. Halfaya** (Georgia-Tech CRNS). She described a particularly robust sensor solution based on HEMT devices fabricated with group III-nitride materials, offering a high resistance to corrosion, humidity and high temperatures. **T. Laroche** (frecnsys SAS) talked about surface-acoustic wave sensors on Langasite substrates for high temperature measurements exceeding 700 °C. He put particular emphasis on explaining a comprehensive model for understanding and computing the electrical response of SAW devices. **X. Zhang** (Beijing University of Science and Technology) described a collection of new approaches for microRNA detection. All of these techniques have the potential to measure microRNA in their typical low concentration with high sensitivity and high specificity. **A. Ionescu** (EPFL) contributed an intriguing talk about how the sensitivity and energy efficiency limits for integrated transducers can be broken with a novel type of Tunnel-FET sensor. These devices offer extreme electrostatic sensitivity of their quantum mechanical band-to-band tunneling current, enabling a future generation of biosensors, gas sensors and imagers operating at voltages as low as 100 mV with unprecedented energy efficiency. The concluding presentation by **J. Leuthold** (ETHZ) described new ways of photonic probing with plasmonic devices. This culminated in the impressive description of an ultra-miniature Mach–Zehnder modulator of only 10 µm in length.

## 6. Three Parallel Sessions

### 6.1. Session 5.1: Photonic Sensing

Session Chair: **Hans Peter Herzig**

The main part of the session was focused on photonic sensing in the mid‐infrared (mid-IR) wavelength range. This activity is strongly driven by the availability of broadly tunable sources, like difference frequency generation (DFG) and external cavity quantum cascade lasers (EC‐QCLs) or the most recent development of diode‐pumped lead salt vertical external cavity surface emitting lasers (VECSELs). The mid-IR is an emerging and important new domain for frontier research. Most complex molecules, such as those found in food, tissue or catalytic compounds, have vibrational spectra in the mid-IR, thus identifiable through mid-IR spectroscopy. Furthermore, the fundamental absorption bands of gas molecules are located in the mid-IR.

A main obstacle for the exploitation of the mid-IR optical window has been a lack of sensitive mid-IR detectors/imaging devices working at room temperature. The first presentation given by **C. Pedersen**, DTU Fotonik (DK) suggested an upconversion technology as a route to circumvent the unavoidable “dark noise” associated with standard mid-IR detectors, thus opening a door to mid-IR, room temperature single-photon spectral imaging and hyper spectral imaging.

Silicon on insulator Vernier devices for high performance photonic sensing in the near- and mid-IR have been presented by **V.M.N Passsaro**, Politecnico di Bari (I). The Vernier effect is widely used in refractive index sensors.

IR-wavelength quantum dot-based sensing has been discussed in the third talk by **V. Woods**, ETH Zurich (CH). Composite semiconductors manufactured by the low-cost, solution-based deposition of colloidally-synthesized semiconductor quantum dots (QDs) are of growing interest.

Laser-based sensing offers several advantages, such as high sensitivity and specificity, large dynamic range, multi-component capability and a lack of pretreatment or preconcentration requirements. The basic principle and various experimental setups have been illustrated by **M.W. Sigrist**, ETH Zurich (CH).

The last three presentations discussed mainly photonic nanostructures and single-photon detection. Films with dimensions smaller than the electron mean free path are essential to measure large resistance changes due to small alterations in thickness or the close proximity of charged species. Simultaneous electrical and plasmonic sensing with gold nanostructures has been demonstrated by **R. Tiefenauer**, ETH Zurich (CH), using ultrathin gold films with incorporated nanoholes.

Zinc oxide nanostructures have received broad attention due to their distinguished performance in electronics, optics, gas sensing and piezoelectronics. The presentation of **S. Rackauskas**, University of Caminas (BR), introduced Zinc oxide tetrapod (ZnO-T) as one of these structures, which consists of four nanowires and is especially interesting for its simple synthesis. ZnO-T is an excellent material for UV sensors.

For many optical sensing and communication applications, including LiDAR, single-molecule spectroscopy and quantum cryptography, detectors with sensitivity at the single-photon level are required. Therefore, the session ended with the presentation of **A. Fiore**, Eindhoven University of Technology (NL), about sensing at the quantum limit, which means at the level of photons. A detector technology based on arrays of superconducting nanowires allows the measurement of the photon number in a pulse in the 1 to 24 photon range.

### 6.2. Session 5.2: Remote and Micropower Sensors

Session Chair: **Assefa M. Melesse**

The first keynote presentation was by **L. Reindl** on power supply for wireless sensor or actuator systems. In this presentation by **L. Reindl**, several concepts for an alternative power supply of wireless sensor or actuator systems were discussed. The advantages of actuators over the ordinary power sources like batteries were presented. The speaker presented an extensive sources of information and direction of the research on wireless sensors or actuators.

The next presentation was by **A.M. Melesse** from Florida International University on sensors and remote sensing applications for understanding coastal and wetland processes. The use of satellite data from Landsat, MoDIS and SeaWIFS was presented in mapping and modeling spatial evapotranspiration (ET), wetland vegetation and also algal blooms before and after tropical storm activities. The use of remote sensing in modeling spatial ET for wetland restoration evaluation was also presented.

The remaining three presenters were from the Polish Academy of Sciences and their research were related and focused on TDR measurements.

**A. Szypłowska**’s talk was on the determination of complex dielectric permittivity spectra from the analysis of electrical signal reflection in transmission lines of various lengths. The speaker stressed that determination of the complex dielectric permittivity frequency spectrum should increase the accuracy of in-situ soil moisture measurements, as well as provide information about other soil properties.

**A. Wilczek** from the Polish Academy of Sciences presented on the impact of the TDR pulse width on the reflection amplitude and its dependence on soil dielectric loss and electrical conductivity. The presented research describes the application of a needle TDR pulse of variable width to measure soil dielectric properties.

**M. Kafarski** also talked about the porous corundum plate sensor for atmospheric water deposits’ TDR measurements. In this presentation, the speaker indicated that of the research was to test the sensor for the atmospheric water deposits intensity measurements and to define its measuring range and detection level.

### 6.3. Session 5.3: Neurosensors

Session Chair: **Patricia A. Broderick**

Neurosensors 5.3 was a lively session, filled with discussion, back and forth questions and answers, and each topic was presented in detail with great aplomb. Each topic was meaningful to micromachines, microelectrodes and nanobiosensors for clinical and preclinical science and medicine. The unique and precise work that was presented during this session was truly translational and, indeed, remarkable.

**S. Lacour** holds the Bertarelli Foundation Chair in Neuroprosthetic Technology at the School of Engineering at the Ecole Polytechnique Fédérale de Lausanne. She received her PhD in Electrical Engineering from INSA de Lyon, France, and completed postdoctoral research at Princeton University (USA) and the University of Cambridge, U.K. **S. Lacour** was the first speaker. Stephanie gave the first keynote speech of the session for one hour. Her presentation was, soft transducers to communicate with the nervous system. Her research focuses on the materials, technology and integration of soft bioelectronic interfaces, including artificial skin, ultra-compliant neural electrodes for *in vitro* platforms, as well as *in vivo* implants. In this speech, there was a description of microelectrode arrays implanted on the surface of the brain and/or within the neural tissue under the brain membranes that surround the brain, under the skull. Stephanie and her group have developed elastic-type bioelectronic interfaces that allow the prosthesis to become more facile, which is a major advance in providing non-ambulating patients the ability to walk. Although this important work is still in progress in animals, the promise of new materials that are more elastic and yet biocompatible provides hope for the paralyzed patient. **S. Lacour** showed a video that allowed an animal to walk. Although the animal was still restrained, the video was impressive! Enabling a paralyzed patient to walk is ground breaking research, and the work is commendable.

**P.A. Broderick** gave the second keynote address for one hour. The title was nanobioimaging: personalized medicine in real life is here. Nanotechnology Meets the Brain. **P.A. Broderick** completed her PhD degree in pharmacology at St. John’s University, College of Arts and Sciences, completed her postdoctoral fellowship at the Albert Einstein College of Medicine/Montefiore Hosp. and completed her Research Associate Position at Cornell University, Department of Neurology, NY. Patricia is a tenured full Professor at the City University of New York School of Medicine and NYU Medical Center and Comprehensive Epilepsy Center. She is Professor at the CUNY Graduate Center in the Department of Biology and Course Director in Pharmacology for the Physicians Assistants Program at the City University of New York School of Medicine, in affiliation with the Harlem Hospital Center, NY, USA. Patricia is widely known for her innovative pioneering work in inventing the BRODERICK PROBE® nanobiosensor for which she has obtained worldwide recognition as an industry expert. This nanobiosensor images chemical and biologic substances within a temporal resolution as low as nanoseconds in real time, *in vivo*, *in vitro* and *in situ* in the intact living brain as a subject is moving about freely. Patricia presented translational work on using this nanobiosensor in epilepsy patients and animals with Parkinson’s disease. Biomarkers for these neurodegenerative brain disorders were revealed and compared to image profiles for brain neurotransmitters in non-diseased parts of brain actually in the same subject, enabling personalized medicine to become a reality clinically. The technology marks the advent of a completely different way to see “how the brain works” and “how to treat the brain that does not work”.

**C.P. Brändli** provided the next speech for one half hour on Neuro-Inspired, Event-Based Vision Sensors. **C.P. Brändli** received his BSc and MSc in interdisciplinary sciences (focus neuroscience and physics) from the ETH Zurich. He conducted his PhD in the lab of T. Delbruck at the Institute of Neuroinformatics, UZH/ETHZ, in Zurich, on the topic of event-based vision sensors. **C.P. Brändli** presented his work on sensors embedded in a micromachine, and the quantum physics was exciting to follow. **C.P. Brändli** also presented his work on vision in the retina based on his micromachine, and he is heavily involved in working with Silicon Valley to move his inventions forward. His startup company is “Insightness”, which he co-founded.

**F. Picollo** gave the fourth speech on diamond-based electrochemical sensor: a multi electrode array for simultaneous detection of quantal exocytic events from neuroendocrine cells. This speech again brought in clinical research showing how graphite sensors, embedded in diamond, can see fluids released from endocrine cells in quantum amounts seeping out of cells. His background is described as PhD in Material Science from the University of Torino in 2011 with a thesis titled “Single crystal diamond micro-fabrication by means of ion beams”. He studied the effects of ion implantation in diamond in order to induce the graphitization of the crystal. He applied this process in order to realize a prototype of a particle detector and a prototype of a biosensor in which buried graphitic structures are the active electrodes. After, he held a post-doc position at the Solid State Physics group of the University of Torino, where his activity was focused on the development of diamond-based cellular sensors. From 2014, he has been the principal investigator of a project called DiNaMo (Diamond Nano Modification) of the National Institute of Nuclear Physics (INFN) devoted to improve the spatial resolution of high energy (MeV) ion beam lithography. **F. Picollo**’s speech also brought about, with the chair and participants, interesting electrochemical principles in which the differences between the allotropes of carbon and their properties affect signals. The chair advised that there might be a signal for adenosine triphosphate in one of the electrochemical signals presented. Suggestions were given to discover a new sensor from this work.

**Y. Leblebici** gave the final speech to round out this great session. His title was: 3D microelectrode arrays for Neuro-Sensing: Read-out circuit design and hybrid 3D integration. **Y. Leblebici** received his PhD degree in electrical and computer engineering from the University of Illinois, Urbana-Champaign (UIUC), in 1990. Since 2002, he has been a Chair Professor at the Swiss Federal Institute of Technology in Lausanne (EPFL) and Director of the Microelectronic Systems Laboratory. His research interests include design of high-speed CMOS digital and mixed-signal integrated circuits, computer-aided design of VLSI systems, intelligent sensor interfaces, modeling and simulation of semiconductor devices, as well as VLSI reliability analysis. He is the coauthor of six textbooks, as well as more than 300 articles published in various journals and conferences. He has been a Fellow of IEEE since 2010, and he has been elected as Distinguished Lecturer of the IEEE Circuits and Systems Society for 2010 to 2011. This speech was related to multi-platform electronics to channel several hundred signals at once. It was extremely interesting and necessary for biosensors, such as **P.A.** **Broderick**’s nanobiosensors for epilepsy patients. There was, however, a feisty debate on the state of the art in this discussed electronic platform. **C. Brandli** thought that this type of complex platform is already available, and **Y. Leblebici** wholeheartedly disagreed.

The session chair would like to thank MDPI in Basel, Switzerland, and Beijing, China, for this fabulous opportunity!

## 7. Session 6: Single-Chip Sensors and Sensor Networks

**Patrick Thomas Eugster**

This session combined presentations describing ultra-miniature sensor systems realized as single-chip solutions and complete networks of miniaturized sensor nodes. In the first talk of this session, **A. de Mello** (ETHZ) introduced droplet-based microfluidic devices, paving the way towards ultra-high throughput experimentation. There is a rapidly growing interest in such lab-on-a-chip technology, in large part driven by concomitant advances in the areas of genomics, proteomics, cellomics, drug discovery, high-throughput screening and diagnostics, with a clearly defined need to perform rapid measurements on ultra-small sample volumes. **C. Schönenberger** (University of Basel) described silicon-nanowire ion-sensitive field-effect transistors, providing for highly-sensitive pH detectors. It is shown how a platform of ion-sensitive sensors can be realized, consisting of differently-functionalized wires, capable of sensing different ions (H^+^, K^+^, Cl^−^, Ca^2+^) with certain selectivity. The following talk by **D. Sallin** (EPFL) introduced a CMOS-compatible photodetector with intrinsic light-to-time conversion for low-light applications, such as bioluminescence. Measurements demonstrate impressive performances down to an intensity of 10^−8^ W/cm^2^ and below. The talk on smart image sensors for metrology applications was contributed by a representative of **E. Franzi** (CSEM). A VLSI implementation of the author’s spaceCoder technology was described, which is a complete vision-based chip specifically tailored for metrology applications. The session part on sensor networks was introduced by **P. Eugster** (Purdue University), who explained a possible progression towards a robust Internet of Things in the case of wireless sensor networks. The challenge is to provide the development of debugging and monitoring solutions for a reliable and secure IoT, including techniques for gathering runtime information on individual sensor nodes’ executions and interactions among nodes, as well as for efficiently compressing such information. The next presentation was given by **T. Watteyne** (UC Berkeley), describing the building of the industrial Internet of Things by transforming the Smart Dust concept into the 6TiSCH standard. Particular attention was given to a concise explanation of IETF 6TiSCH, the working group that combines the performance of industrial low-power wireless with the ease of use of IPv6. **C.-Y. Wen** (National Chung Hsing University) explained an energy-efficient scheduling method with compressed sensing for distributed target tracking. In this method, a two-level hierarchical wireless sensor network is designed for cooperative target tracking via compressing information. The final contribution by Jean-Dominique Decotignie (CSEM) summarized his experience and lessons learned in designing various wireless sensor networks for real-time operation with low power consumption, realized, for example, on glaciers, in buildings, on trains and on planes. Among other things, the author dispelled seven myths (e.g., “signal strength is a function of distance”) about wireless sensor network design.

